# Reduced Activity in the Right Inferior Frontal Gyrus in Elderly *APOE*-E4 Carriers during a Verbal Fluency Task

**DOI:** 10.3389/fnhum.2017.00046

**Published:** 2017-02-06

**Authors:** Andrea Katzorke, Julia B. M. Zeller, Laura D. Müller, Martin Lauer, Thomas Polak, Andreas Reif, Jürgen Deckert, Martin J. Herrmann

**Affiliations:** ^1^Department of Psychiatry, Psychosomatics and Psychotherapy, University Clinic WürzburgWürzburg, Germany; ^2^Department of Psychiatry, Psychosomatic Medicine and Psychotherapy, University Hospital FrankfurtFrankfurt am Main, Germany

**Keywords:** near-infrared spectroscopy, verbal fluency task, elderly, apolipoprotein-E4, Alzheimer’s disease

## Abstract

Apolipoprotein-E4 (*APOE*-E4) is a major genetic risk factor for developing Alzheimer’s disease (AD). The verbal fluency task (VFT), especially the subtask category fluency, has shown to provide a good discrimination between cognitively normal controls and subjects with AD. Interestingly, *APOE*-E4 seems to have no effect on the behavioral performance during a VFT in healthy elderly. Thus, the purpose of the present study was to reveal possible compensation mechanisms by investigating the effect of *APOE*-E4 on the hemodynamic response in non-demented elderly during a VFT by using functional near-infrared spectroscopy (fNIRS). We compared performance and hemodynamic response of high risk *APOE*-E4/E4, -E3/E4 carriers with neutral *APOE*-E3/E3 non-demented subjects (*N* = 288; 70–77 years). No difference in performance was found. *APOE*-E4/E4, -E3/E4 carriers had a decreased hemodynamic response in the right inferior frontal junction (IFJ) with a corresponding higher response in the left middle frontal gyrus (MFG) during category fluency. Performance was correlated with the hemodynamic response in the MFG. We assume a compensation of decreased IFJ brain activation by utilizing the MFG during category fluency and thus resulting in no behavioral differences between *APOE*-groups during the performance of a VFT.

## Introduction

A great deal of worry of elderly is age related dementia, with its most common form of Alzheimer’s disease (AD; 60%–70%; World Health Organization, 2015). A major risk factor for developing AD is a specific isoform of the lipid transporting protein Apolipoprotein E (APOE; Corder et al., 1993, 1994; Roses, 1996; Bertram et al., 2007). *APOE4* contributes to 20%–25% of the heritable component in late-onset AD susceptibility (Lambert et al., 2013; Jun et al., 2016). There are three major *APOE4* isoforms in humans: *APOE*-E2, *APOE-E3* and *APOE*-E4. The mean age of onset becomes lower and the risk of developing AD increases for each additional *APOE-*E4 allele carried, whereas *APOE-*E2 seems to be protective regarding AD when considering *APOE-*E3 as neutral (Corder et al., 1993, 1994).

In functional imaging studies on aging, AD as well as *APOE-*E4 have shown to be associated with changes in functional brain activation (Smith et al., 2002; Herrmann et al., 2008; Steffener et al., 2009; Grady, 2012; Kahlaoui et al., 2012; Trachtenberg et al., 2012). These changes are often interpreted as an (either *attempted* or *successful*) compensation mechanism (Grady, 2012). For example, it has been shown that age-related limitations like decreases in the regional gray matter volume and as a consequence a decreased efficient use of a primary brain network is associated with the increased recruitment of other regions (Steffener et al., 2009). Based on its relationship to the behavioral performance this can be considered as a successful compensation (Steffener et al., 2009). Moreover, reduced activity in the prefrontal cortex could be found for older adults compared to younger adults during a verbal fluency task (VFT; Kahlaoui et al., 2012). Similarly, participants with AD compared to healthy controls that are similar in age and sex had a reduced functional activation of prefrontal regions during a VFT (Herrmann et al., 2008). By reviewing literature that investigated the effect of *APOE-*E4 on the blood oxygen level dependent (BOLD) response, no clear pattern regarding the direction and location of functional brain activity differences could be found (Trachtenberg et al., 2012). The pattern could be due to the high variability of the experimental design of the studies that were reviewed (Trachtenberg et al., 2012). To our best knowledge, the one and only study that investigated the effect of *APOE-*E4 on functional imaging during a VFT found an increased activity in the parietal region for *APOE-*E4 carriers (Smith et al., 2002). However, this study had a small sample size (*N* = 38) and thus possible effects in frontal regions, which are utilized during a VFT, could have been undetectable.

A recent integrative model to account for brain activation changes in elderly is the Scaffolding Theory of Cognitive Aging (STAC; Park and Reuter-Lorenz, 2009). This model was extended to STAC-r, taking life-course factors additionally into account (Reuter-Lorenz and Park, 2014). Reuter-Lorenz and Park (2014) suggest that challenges with aging like decreases in brain structure size are compensated by an adaptive brain engaging in compensatory scaffolding. This compensatory scaffolding ability can be enhanced (“neural resource enrichment”) as well as depleted (“neural resource depletion”) by life-course factors and genetic endowment (Reuter-Lorenz and Park, 2014). For example, they assume *APOE-*E4 to have an exceedingly powerful depletion effect on cognition (Reuter-Lorenz and Park, 2014). The ability of the brain to engage in scaffolding in dependence of life-course factors and/or genetic endowments (e.g., emerging as *attempted* rather than *successful* compensational scaffolding) could be detected by functional imaging.

In this study, we chose to examine scaffolding processes by functional brain activation alterations of* APOE*-E4 carriers during a VFT because of its discriminative characteristics between AD and healthy controls as described later. The VFT typically consists of a category and a letter task. During a VFT, subjects are usually instructed to name as many words as possible from a specific category or beginning with a specific letter. It measures the rapid access to phonological and semantic information, utilizing executive functions (Shao et al., 2014). Whereas category fluency is supposed to evaluate the functioning of the semantic memory (Henry et al., 2004), letter fluency requires working memory processes (Shao et al., 2014). Functional imaging with functional near-infrared spectroscopy (fNIRS) has shown that bilateral inferior frontal junctions (IFJ) were utilized during letter as well as category VFT (Heinzel et al., 2013, 2015). This finding is consistent with the link of the inferior frontal gyrus (IFG) to phonological and semantic operations (Costafreda et al., 2006).

The performance during a VFT provides a good discrimination between cognitively normal controls and subjects with AD (Monsch et al., 1992; Henry et al., 2004). In line with impaired access to the semantic memory in AD (Adlam et al., 2006), category fluency (sensitivity 100%, specificity 92.5%) demonstrated a better discrimination between subjects with AD and normal controls than letter fluency (sensitivity 89%, specificity 85%; Monsch et al., 1992). A decreased performance in category fluency is even detectable approximately 5.4 years before developing AD and also in elderly with mild cognitive impairment (MCI; Adlam et al., 2006; Clark et al., 2009). Venneri et al. (2015) even proposed a paradigm shift from the recognition of the episodic memory as main symptom of prodromal AD to measurements of the semantic memory, since the semantic memory remains more stable across the lifespan compared to the episodic memory. Thus, measurements of the semantic memory can be highly discriminative between healthy and pathological aging (Venneri et al., 2015). By comparing *APOE-*E4 carriers and non-carriers in the performance of the VFT no difference could be found for middle-aged and elderly participants (Blair et al., 2005; Schiepers et al., 2012) or even a better performance for middle-aged and elderly *APOE-*E4 carriers (Marioni et al., 2016).

Several studies also indicate structural and functional alterations in *APOE*-E4 carriers over the lifetime. Structurally, *APOE*-E4 carriers were found to have a reduced gray matter volume in temporal areas and increased volume in frontal areas at birth and in middle-age (Dean et al., 2014). Despite an increased volume in frontal areas at birth and in middle-aged, healthy *APOE*-E4 carriers (age: 48–75 years) have shown to have an enhanced thinning of frontal and temporal regions compared to non-carriers with increasing age (Espeseth et al., 2008). Furthermore, in elderly *APOE*-E4 carriers with MCI, a prodromal stage of AD, a reduced gray matter volume of the medial temporal as well as the right IFG was found compared to non-carriers with MCI (Thomann et al., 2008). Functionally, *APOE*-E4 carriers compared to non-carriers have shown to have a higher task-induced activity in frontal and temporal regions in younger ages, but a decreased activity in higher ages although a similar behavioral performance during a memory encoding task (Filippini et al., 2011).

For the VFT there is no study to date that investigated the effect of *APOE-*E4 isoforms on functional imaging in elderly over 65 years. Functional imaging during a VFT in elderly could help us understand why the performance of the VFT differs between elderly before developing AD and normal controls but not between carriers of different *APOE-*E4 isoforms. fNIRS provides a useful method to measure regional brain tissue oxygenation during speech tasks because it is less sensitive to motion artifacts compared to functional magnetic resonance imaging (Fallgatter et al., 2004). Furthermore, fNIRS is easy to administer and it is less expensive and has a higher temporal resolution compared to functional magnetic resonance imaging (Cui et al., 2011). FNIRS measures relative changes of oxyhemoglobin [O_2_Hb] as well as deoxyhemoglobin [HHb]. Since [HHb] is a valid parameter for brain activity when using fNIRS and [O_2_Hb] has shown to be influenced by noise (Obrig and Villringer, 2003), we decided to discuss only [HHb] in the discussion but to report [O_2_Hb] as well as [HHb] in the “Results” Section. The purpose of our study was to investigate differences between *APOE*-groups (risk: E4/E4, E3/E4 vs. neutral: E3/E3) in hemodynamic response measured by fNIRS during VFT in non-demented subjects. We compared *APOE*-E4/E4, -E3/E4 to *APOE*-E3/E3 but not to carriers of the *APOE*-E2 allele because the relationship between the *APOE-*E2 isoform and AD neuropathology is ambiguous and not thoroughly investigated to date (Berlau et al., 2009). We hypothesized that: (1) VFT performance does not differ between *APOE*-groups; (2) *APOE-*E4/E4, -E3/E4 exhibit a decreased activation in IFJ compared to *APOE-*E3/E3; (3) this decreased activation is compensated by utilizing other regions; and (4) in significantly differing channels, hemodynamic response is moderated by *APOE*-groups in dependence of performance.

## Materials and Methods

### Subjects

In this study, data of 604 volunteers were collected in the context of the first data acquisition of a longitudinal study (“Vogel-Studie”) examining early diagnosis of dementia (Polak et al., submitted). The study was approved by the local ethics committee of the Medical Faculty of the University Clinics Würzburg (internal number 23/11) and complied with the declaration of Helsinki. Every participant signed a written informed consent after receiving detailed information of the study. Vulnerable populations were not involved.

**Figure 1:**
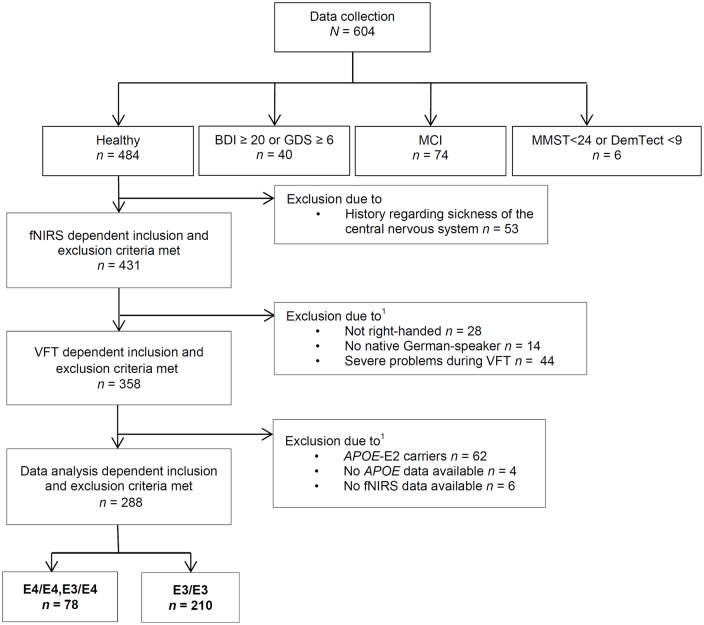
**Course of exclusion for data analysis.** VFT, verbal fluency task; MCI, mild cognitive impairment; *APOE4*, Apolipoprotein-E4; fNIRS, functional near-infrared spectroscopy; BDI, Beck’s Depresson Inventory-II; GDS, geriatric depression screening scale; MMST, Mini-Mental-Status-Test; DemTect, dementia detecting screening test; ^1^some subjects were excluded due to more than one reason.

For data collection (see Figure 1 for the Flow Chart) we included participants who were free of any severe psychiatric, neurological and internal disease for the last 12 months and did not suffer from severe and uncorrected impaired vision or hearing on the day of the examination. For data analysis, subjects that had a history of a central nervous system disease were excluded.

We also excluded participants with a suspected medium or severe depression, MCI or a positive dementia screening. Suspected medium or severe depression was defined as a Beck Depression Inventory-II (BDI-II) score equal to or above 20; or a geriatric depression screening scale (GDS) score equal to or above 6 (Yesavage et al., 1982; Beck et al., 1996; Gauggel and Birkner, 1999; Hautzinger et al., 2006). A positive dementia screening was defined by a Mini-Mental-Status-Test (MMST) score below 24 or a DemTect score below 9 (Folstein et al., 1975; Kalbe et al., 2004). According to the criteria for suspected MCI by Portet et al. (2006), participants with subjective cognitive impairment, noticeable cognitive problems in clinical examination, without a conspicuous dementia screening and no suspected medium or severe depression and without impairment of daily activities (Bayer-Activities of Daily Living Scale <2.1; Hindmarch et al., 1998) were defined as MCI and not further analyzed in this study.

Furthermore, only right-handed native German speakers and participants without severe problems during the performance of the VFT (e.g., technical problems, misunderstanding of the task etc.) were included in these analyses. Some subjects were excluded from analysis because fNIRS- and *APOE* data were not available. We excluded *APOE*-E2 carriers because the relationship between the *APOE-*E2 isoform and AD neuropathology is ambiguous and not thoroughly investigated as well because of the small amount of *APOE-*E2/E3 and -E2/E2 positive participants (*n* = 47) resulting in a low power (Berlau et al., 2009).

In summary, we thus analyzed in this study data of 288 right-handed subjects. 210 subjects (103 female, 107 male) were carriers of the neutral gene variant *APOE-*E3/E3, seven subjects (two female, five male) were homozygotes of the risk gene variant *APOE-*E4/E4 and 71 subjects (40 female, 31 male) were carriers of a risk allele as well as a neutral allele (*APOE*-E3/E4). Because of the small sample size of *APOE-*E4 homozygotes, we formed a joint group of *APOE-*E4/E4 carriers and *APOE*-E3/E4 carriers. Sample characterization is presented in Table 1.

**Table 1:** **Sample characterization**.

	E3/E3 (*n* = 210)		E4/E4, E3/E4 (*n* = 78)	
	*M*	*SD*	*M*	*SD*	*t*	*df*	*p*
Age	73.89	1.55	73.67	1.62	1.08	286	0.282
Education (years)	10.88	3.60	11.05	3.88	−0.34	284	0.733
DemTect	16.35	1.87	16.09	2.40	0.87	113.6^a^	0.384
MMST	29.20	0.97	29.37	0.88	−1.40	286	0.161
BDI-2	5.49	4.38	5.29	4.13	0.34	286	0.733
GDS	1.04	1.23	1.06	1.38	−0.13	286	0.900
B-ADL	1.39	0.51	1.43	0.56	−0.59	286	0.553

*Note: ^a^Correction for heteroscedasticity*.

### Verbal Fluency Task

We used three different kinds of subtasks to measure the verbal fluency of the subjects like reported elsewhere (Herrmann et al., 2008). In the letter version subjects were instructed to pronounce as many German nouns as possible beginning with the letters “A”, “F” and “S” without using proper names and repetitions. In the category version subjects were asked to list German nouns belonging to the categories “animals”, “fruits” and “flowers”. The third subtask was applied to control for hemodynamic response changes induced by speech. Subjects were asked to repeat all days of the week at a moderate speed in a consecutive manner. The tasks were presented as a block design with 60-s blocks consisting of 30 s of activation and 30 s of rest in each condition. Behavioral performance was measured by recording the number of correct verbal responses. Furthermore, the subjects were instructed to avoid speaking during the resting phases, to close their eyes and to avoid movements with their head during the whole VFT. The stimuli were presented by verbal instructions.

### Functional Near-Infrared Spectroscopy

A 52-channel continuous wave system (ETG-4000, Hitachi Medical Corporation, Tokyo, Japan) was used. The ETG-4000 applies near-infrared light at two different wavelengths (*M* = 695 nm, *SD* = 20 nm and *M* = 830 nm, *SD* = 20 nm) to measure relative concentration changes of [O_2_Hb] and [HHb] in the vascular system of the cerebral cortex. The sampling rate was 10 Hz. We adjusted a 3 × 11 probeset including 17 laser-diodes and 16 photo detectors with elastic straps on the forehead of the subjects. With regard to the 10–20 electrode system, we placed the middle probe of the lowermost row over FPz (American Electroencephalographic Society, 1994).

For classification of channels to anatomical regions, we used virtual registration results (Tsuzuki et al., 2007). These results are based on a technique of simulating different head sizes and shapes to get estimated stereotactic brain coordinate data of NIRS channels (Tsuzuki et al., 2007). Regions were identified by using the most probable anatomical label (Tzourio-Mazoyer et al., 2002).

### *APOE* Genotyping

Blood was taken on the day of examination. *APOE* was genotyped like described by Hixson and Vernier (1990). In short, we used polymerase chain reaction (PCR) with the forward primer 5′-TAA GCT TGG CAC GGC TGT CCA AGG A- 3′ and the reverse primer 5′- ACA GAA TTC GCC CCG GCC TGG TAC ACT GCC- 3′. In a total volume of 25 μl the PCR mixture contained 2.5 μl Goldstar, 1 μl 25 mM MgCl2, 1 μl 2.5 mM Nuk each, 1 μl (10 pmol/μl) of each primer, 0.3 μl Taq, 0.8 μl DMSO, 16.4 μl H_2_O and 50 ng of genomic DNA. Our cycling conditions were 95°C for 5 min, 45 s at 95°C, 45 s at 65.2°C and 45 s at 72°C for 38 cycles, 5 min at 72° and a pause at 10°C. The PCR product with a fragment size of 244 bp was digested using HinP1I (New England Biolabs, Frankfurt am Main, Germany) for 2 h at 37°C and then migrated on a peqGOLD MoSieve Agarose MS-500.

### Data Analyses

#### Task Performance

The number of correct words in each subtask was averaged and *APOE*-groups (E3/E3 vs. E4/E4, E3/E4) were compared using an independent *t*-test.

#### Functional Near-Infrared Spectroscopy

**Figure 2:**
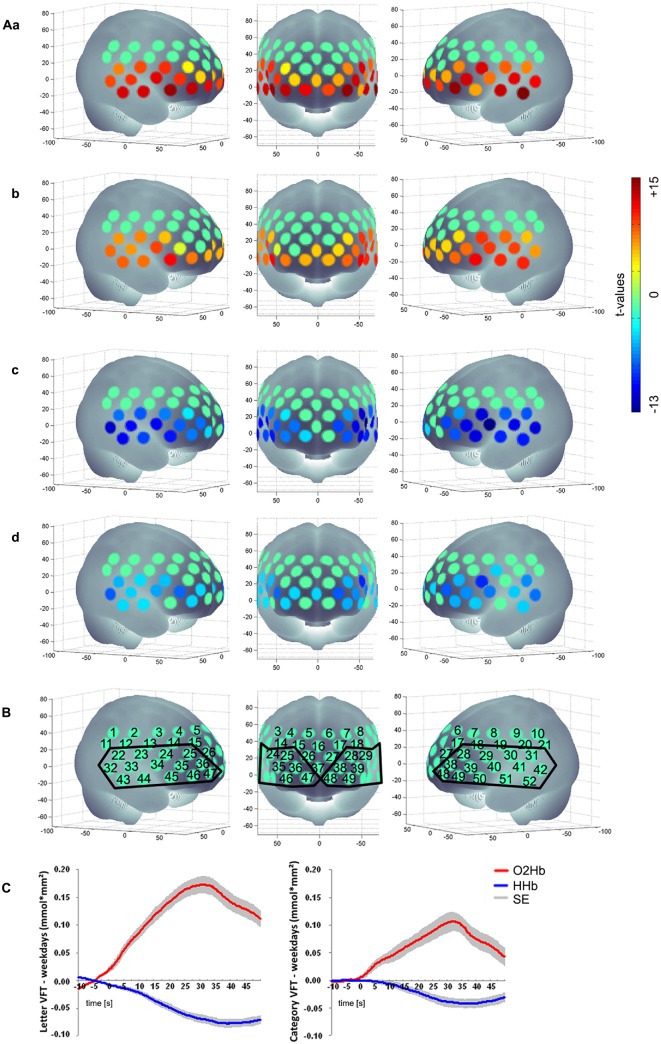
**(Aa)** Letter [O_2_Hb], **(b)** Category [O_2_Hb], **(c)** Letter [HHb], **(d)** Category [HHb]; **(B)** Numerical labeling of projected fNIRS and defined ROI based on the activation pattern found; **(C)** mean hemodynamic response of the active tasks (left: letter VFT; right: category VFT) minus the control condition (weekdays) for all subjects over the ROI as shown in **(B)**; baseline [10 s], task [30 s], post-measurement [20 s]; positive values imply an increased hemodynamic response during task performance compared to the control condition regarding [O_2_Hb]; for [HHb] negative values imply an increased hemodynamic response during task performance compared to the control condition; non-significant channels are characterized by *t*-value of 0, presented in a green color; SE, standard error.

We excluded participants because of either severe problems in understanding and performing the task or because of poor signals/artifacts identified by visual inspection (summarized as “severe problems during VFT” in Figure 1). For data analysis we applied a cosine filter to remove slow drifts and a moving average filter with a time window of 5 s to eliminate high frequency portion. Furthermore, we applied a common average reference (CAR) to reduce systemic artifacts (Bauernfeind et al., 2013). We used MatLab 7.7.0 (MathWorks Inc., Natick, MA, USA) to calculate the relative changes in mean activation and standard deviation per participant, per channel, per task version and per phase (baseline [10 s], task [30 s], post-measurement [20 s]). Based on the suggestion by Schroeter et al. (2003), we calculated effect sizes to account for anatomically inter- and intrasubject differences in the differential pathlength factor. We calculated the effect size by subtracting the arithmetic mean of the baseline from the mean activation during the task performance and finally dividing the result by the standard deviation of the baseline. Subsequently, the effect size of the control condition was subtracted from the effect size of the task for each person and each task due to correction for speech-induced activity. Moreover we used the Dubey/Armitage-Parmar procedure (DAP) for each task to identify significantly activated channels during VFT. DAP is a procedure that takes the high spatial correlation of fNIRS data into account (see Figure 2; Sankoh et al., 1997).

Differences in relative changes of hemodynamic response between *APOE*-groups were compared by *t*-tests for each channel. Multiple comparison correction was applied by using false discovery rate (FDR; one-tailed, *q* [maximum of false positives on average] was set to 0.05; Singh and Dan, 2006).

To figure out if differences found in hemodynamic response are associated with an *attempted* or *successful* compensation mechanism, we first calculated if there is a general association between performance and hemodynamic response by a partial correlation with the control variables age, gender and years of education in channels where differences between *APOE*-groups were found (one-tailed, Bonferroni correction). Second, to test if the hemodynamic response is moderated by *APOE*-group in dependence of performance we compared the regression coefficients between the *APOE-*groups by applying the procedure as described by the Statistical Consulting Group (2016). Again, we controlled for age, gender and years of education and a Bonferroni correction was applied.

## Results

### Task Performance

There was no significant difference in task performance between groups neither in letter condition (E3/E3: *M* = 6.76, *SD* = 2.25; E4/E4, E3/E4: *M* = 6.97, *SD* = 2.50, *t*_(286)_ = −0.69, *ns*) nor in category condition (E3/E3: *M* = 11.01, *SD* = 2.36; E4/E4;E3/E4: *M* = 11.11, *SD* = 2.54, *t*_(286)_ = −0.31, *ns*).

### Functional Near-Infrared Spectroscopy

#### Activation Pattern

We found a significant activation of 29 channels for [O_2_Hb] and of 27 channels for [HHb] for letter condition and for category condition a significant activation of 27 channels for [O_2_Hb] and of 24 channels for [HHb] when contrasting the tasks against the control task. Figure 2 shows activation pattern for both tasks in bilateral frontal regions.

#### ApoE-Group Comparison

**Figure 3:**
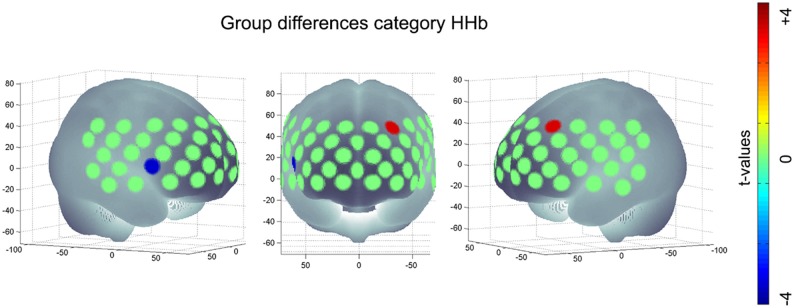
**Significant group difference of risk allele carriers (*APOE-E4/E4, -E3/E4*) compared to neutral group (*APOE-E3/E3*) during category task HHb; positive values imply an increased hemodynamic response (i.e., a higher negative value for [HHb]) in subjects with *E4/E4, -E3/E4* compared to *E3/E3*; negative values imply a decreased hemodynamic response (i.e., a more positive value for [HHb]) in subjects with *APOE-E4/E4, -E3/E4* compared to subjects with *APOE-E3/E3***.

Regarding [HHb] during category task, subjects with *APOE-*E4/E4 or -E3/E4 (*M* = −1.74, *SD* = 11.96) revealed a significant more negative change, i.e., a significant higher increase in hemodynamic response compared to *APOE-*E3/E3 (*M* = 2.87, *SD* = 9.83, *t*_(286)_ = 3.33, *p* < 0.001, FDR-correction) in the medial frontal gyrus (channel #7). On the other hand, subjects with *APOE-*E4/E4 or -E3/E4 (*M* = 0.20, *SD* = 7.23) had a lower hemodynamic response in the IFJ (channel #34) compared to *APOE-*E3/E3 (*M* = −3.03, *SD* = 7.10, *t*_(286)_ = −3.41, *p* < 0.001, FDR-correction; see Figure 3). No significant group differences were found for letter [O_2_Hb], letter [HHb] or category [O_2_Hb].

#### Association Between Performance and Hemodynamic Response

We calculated a partial correlation with the control variables age, gender and years of education in channels with significant *APOE4*-group differences (channel #7 and #34 regarding [HHb] during category task). We found a significant negative correlation between performance and hemodynamic response in channel #7 (*r* = −0.140, *p* = 0.009), indicating a positive association between performance and hemodynamic response. No significant association was found for channel #34 and hemodynamic response (*r* = 0.003, *ns*).

We compared the regression coefficients between both groups and controlled for age, gender and years of education. We found no significant difference for both groups in the prediction of the hemodynamic response by performance, neither for channel #7, nor for channel #34.

## Discussion

In this study we compared the performance and changes in hemodynamic response during a VFT between *APOE*-groups (*APOE-*E4/E4, -E3/E4 vs. *APOE-*E3/E3). In line with previous results, VFT performance did not differ between *APOE*-groups (Blair et al., 2005; Packard et al., 2007; Wisdom et al., 2011; Schiepers et al., 2012). Looking at functional imaging, we found a significantly decreased activation in the IFJ for *APOE-*E4/E4, -E3/E4 compared to *APOE-*E3/E3 for category fluency. In contrast, in the middle frontal gyrus (MFG) was a significanty increased activation for *APOE-*E4/E4, -E3/E4 compared to *APOE-*E3/E3 for category fluency. Furthermore, we found a significant negative correlation between performance and hemodynamic response in the MFG, indicating a positive association between performance and hemodynamic response. No association was found for performance and hemodynamic response in IFJ. However, the association of performance and hemodynamic response in IFJ and MFG was not moderated by *APOE*-group. Interestingly, we found the frontotemporal region (including the IFJ) to be significantly activated during the VFT but not the MFG.

The MFG is a part of the frontoparietal control system. The frontoparietal control system is linked to executive functions, such as flexibility and working memory (Niendam et al., 2012). Cole et al. (2014) assume that by a robust control system even after brain lesions humans may have improved outcomes by facilitating recovery in a goal-directed manner. For example, it has been shown that the frontoparietal control system is important for speech recovery after brain lesions (Brownsett et al., 2014). The positive association between performance and hemodynamic response in the MFG could thus reflect a successful recruitment of the control system in accordance with the assumption of a compensatory scaffolding mechanism as proposed by Reuter-Lorenz and Park (2014).

Nevertheless, it is surprising that no association between performance and hemodynamic response was found for regions that utilized during the VFT according to literature and our results utilized during the VFT (frontotemporal region including the IFJ; Herrmann et al., 2008; Tupak et al., 2012; Heinzel et al., 2013, 2015). In contrast to our results, Metzger et al. (2016) found a negative correlation of performance and hemodynamic response in the Broca region (which is part of the IFJ). In line with our findings, his analysis also revealed a positive correlation in the parietal cortex and the MFG (which he referred to as dorsolateral prefrontal cortex) in patients with AD during a VFT. Metzger et al. (2016) argued that his results indicate an inverse relationship of structures within the frontoparietal control system and language-specialized areas.

The increased activity of the MFG and the corresponding decreased activity in the IFJ for *APOE-*E4/E4, -E3/E4 carriers could reflect a compensation mechanism. Previous studies found the brain activity in the IFJ during a VFT to be negatively correlated with age (Heinzel et al., 2013, 2015) and decreased in patients with AD (Herrmann et al., 2008; Schroeter et al., 2011). Furthermore, Heinzel et al. (2013, 2015) did not only find age negatively correlated to activity in the IFJ but also to be positively correlated to activity in the MFG and supramarginal gyri. These results were interpreted in terms of a compensatory increased recruitment of the frontoparietal control system. However, we did not find group differences when comparing regression coefficients in these regions. A possible explanation could be that both groups have similar additional recruitment strategies in these regions but *APOE-*E4/E4, -E3/E4 carriers have to apply these additional recruitment strategies to an greater extent compared to *APOE-*E3/E3 carriers. In line with that, we found a significant positive association in the MFG for both groups.

Though, despite thorough measurement and elimination of artifacts by using CAR and visual inspection, effect sizes and contrasting of the task with a control condition (see “Functional Near-Infrared Spectroscopy” Section), we cannot completely exclude the possibility that some artifacts, e.g., motion artifacts, could have had an effect on our results. Even so, our results are in line with findings of previous studies (Herrmann et al., 2008; Schroeter et al., 2011; Heinzel et al., 2013, 2015; Metzger et al., 2016). Hence, artifacts are unlikely to cause our results.

In summary, our present study has shown that in non-demented high-risk *APOE-*E4 subjects, a decrease of hemodynamic response in the IFJ and an increase of hemodynamic response in the MFG could be found. Moreover, performance was positively correlated with the hemodynamic response in the MFG. The recruitment of the MFG could represent a compensation mechanism that should be further investigated.

## Author Contributions

All authors approved the final version to be published and agreed to be accountable for all aspects of the work in ensuring that questions related to the accuracy or integrity of any part of the work are appropriately investigated and resolved. Furthermore, AK contributed to the analysis of the work and drafted the article. JBMZ and LDM contributed to the acquisition of the work and revised the article. ML and TP contributed to the interpretation and revised the article. AR contributed to the interpretation of the work and revised the article. JD contributed to the conception of the work and revised the article. MJH contributed to the conception of the work and drafted the article.

## Conflict of Interest Statement

The authors declare that the research was conducted in the absence of any commercial or financial relationships that could be construed as a potential conflict of interest.
